# The Effectiveness of Pediatric Central Venous Access Device Surveillance and Rounding Team: A Single-Center Study

**DOI:** 10.7759/cureus.57855

**Published:** 2024-04-08

**Authors:** Shojiro Hanaki, Shuichi Katayama, Soichi Nakada

**Affiliations:** 1 Division of Pediatric Surgery, Department of General Surgery, Kurashiki Central Hospital, Kurashiki, JPN

**Keywords:** central venous line, central venous access device, bedside rounding, children's, complication of central line, central venous catheter (cvc), central venous access

## Abstract

Introduction

Central venous access devices (CVADs) are indispensable in the management of pediatric cancer patients, offering vital access to treatment. Yet, complications related to CVADs, such as infections, thrombosis, and dislocations, pose significant risks, potentially leading to prolonged hospitalization, intensive care unit admission, or even mortality. To address these challenges, our hospital established a pediatric CVAD surveillance and rounding team to improve the management and care of pediatric patients with CVADs.

Materials and methods

This single-center retrospective study evaluated the impact of the pediatric CVAD surveillance and rounding team on the management of pediatric oncology patients with CVADs at Kurashiki Central Hospital, Kurashiki, Japan. We included pediatric cancer patients under 18 years of age who underwent CVAD placement from January 2018 to December 2022. The team conducted weekly rounds focusing on a comprehensive checklist to ensure optimal CVAD care. We compared the incidence of catheter-related complications before and after the establishment of the rounding team using the Student’s t-test and Fisher’s exact test.

Results

The study encompassed 28 patients before and 39 after the implementation of the surveillance rounds. Significant reductions were observed in the number of dislocations (from 28.6% to 0%, p = 0.001) and local infections (from 17.9% to 2.6%, p = 0.04). While the decreases in thrombosis, catheter breakage/rupture, and catheter-related bloodstream infections (CRBSIs) did not reach statistical significance, they suggest a favorable trend toward enhanced management of CVADs.

Conclusions

The establishment of a pediatric CVAD surveillance and rounding team significantly reduced the incidence of dislocations and local infections among pediatric cancer patients with CVADs. This multidisciplinary team approach highlights the importance of continuous surveillance, teamwork, and education in enhancing the quality of CVAD care, contributing to safer patient outcomes and emphasizing the need for continuous improvement in pediatric CVAD management.

## Introduction

Central venous access devices (CVADs) are pivotal in the treatment of pediatric cancer patients, providing essential access to chemotherapy, nutrition, and blood products. However, the use of CVADs is not without risk: complications such as infections, thrombosis, and catheter dislocation can lead to significant morbidity, extending hospital stays, necessitating intensive care, or even resulting in mortality [1]. The challenge of managing these devices is exacerbated in the pediatric population, where size, growth, and activity levels, as well as the psychological impact on young patients and their families, introduce unique complexities [2]. This risk is further complicated by the higher risk of complications, such as catheter self-removal, particularly in very young or non-compliant children [2].

Recognizing these challenges, Kurashiki Central Hospital, Kurashiki, Japan, initiated a pioneering approach by September 1, 2020, by establishing a pediatric CVAD surveillance and rounding team. This multidisciplinary team, consisting of pediatric surgeons, pediatricians, pediatric nurses, pediatric nurse practitioners, and ward nursery teachers, was designed to oversee the comprehensive care of pediatric patients with CVADs. This approach aims to mitigate the risk of complications through regular assessments based on a detailed checklist of nine specific points, ensuring the highest standards of CVAD care.

The role of each team member is strategically defined to address the multifaceted aspects of CVAD management. Pediatric surgeons leading the team oversee the surgical management of CVADs, ensuring technical excellence in placement and interventions. Pediatricians provide an additional layer of medical oversight, addressing comprehensive patient care and potential complications. Pediatric nurses and nurse practitioners are crucial to daily management, focusing on signs of infection, line maintenance, and family education. Additionally, ward nursery teachers offer unique support, concentrating on the psychological well-being and comfort of the children, which is essential to fostering a supportive care environment that promotes calmness and cooperation during procedures and rounds.

This collaborative framework serves as the cornerstone of our approach, seeking to enhance CVAD management in pediatric patients by leveraging the diverse expertise of the team members. It highlights the hospital’s proactive stance on improving patient care through dedicated, multidisciplinary efforts.

## Materials and methods

This retrospective study aimed to evaluate the management status of pediatric patients with CVADs in our hospital and to assess the effectiveness of the pediatric CVAD surveillance and rounding team’s management and observation. The subjects were pediatric cancer patients (<18 years old) who underwent CVAD placement (Hickman-Broviac® [C.R.Bard, Central Avenue Murray Hill, New Jersey, USA]) and were hospitalized in our pediatric ward from January 2018 to December 2022. The study was approved by the institutional review board (IRB) of Kurashiki Central Hospital (medical ethics committee) on November 23, 2023 (approval number: 3780), with patients being offered the opportunity to opt out.

The surgical procedure is performed as follows: the procedure begins under general anesthesia, and catheterization is performed via either the left or right internal jugular or subclavian vein, with the external outlet positioned 3-4 cm below the clavicle along the midline of the clavicle. The cuff is placed 2 cm away from the outlet in the subcutaneous tunnel. The outer portion of the catheter is sutured and secured to the skin with three non-absorbable sutures. The incision site is covered with a dressing material, and both the neck and chest incision sites are compressed. Compression is released the day after surgery.

The rounding method included a weekly comprehensive rounding, extending from admission to discharge, in which nine points, including dressing, fixation, and skin condition, were assessed and shared among the multidisciplinary team, as listed in Table 1. Each patient’s condition was evaluated consistently every week throughout their entire hospital stay. During team rounds, we endeavored to enhance communication with patients and their caregivers regarding CVAD management. Additionally, pediatric residents attending the rounds had the opportunity to observe a CVAD catheter placement procedure, thereby enhancing their understanding of its structure. Following the evaluation, any identified issues were immediately addressed, and the effectiveness of these interventions was re-evaluated the following week, ensuring continuous improvement in CVAD care and management.

**Table 1:** Weekly checklist: including dressing, fixation, and skin condition among 9 items CVAD, central venous access device

Criteria	Description
CVAD insertion site coverage	The CVAD insertion site is adequately covered to prevent infection and reduce the risk of catheter displacement.
Fixation at insertion site	There is no displacement in the fixation at the insertion site, ensuring the catheter remains securely in place.
Fixation below insertion site	There is no displacement in the fixation below the insertion site, further securing the catheter's position.
Catheter security	The catheter is secured in such a manner as to form a bend without being pulled, maintaining its function and reducing discomfort.
Catheter movement	The catheter remains in place without being pulled when the patient lies down, sits up, or moves their arms and shoulders, indicating proper fixation and flexibility.
Distance before suture removal	For patients prior to suture removal, the distance between the insertion site and the fixation thread is maintained, ensuring stability.
Cuff position	The position of the cuff can be felt with the fingers, with no protrusion of the cuff, indicating proper placement and reduced risk of infection.
Site condition	There is no redness, itching, or discharge around the insertion site, signs of good catheter care and absence of infection.
Fixation stitches before suture removal	For patients prior to suture removal, fixation stitches are left in place and secured to the skin, preventing premature movement or removal.

A retrospective comparison was conducted between the periods before and after the initiation of pediatric CVAD surveillance and rounding with respect to catheter-related bloodstream infection (CRBSI), local infection, thrombosis, dislocation, and catheter breakage/rupture. No changes in surgical technique or management occurred during the observation period.

Local infections, such as phlebitis, infections at the exit site, or tunnel infections, were defined as conditions in which the exit site culture tested positive within 2 cm of the CVAD track and exit site or that presented with erythema, purulent discharge, and tenderness [3]. CRBSI was defined according to the criteria of the “2009 Update by the Infectious Diseases Society of America,” similar to the definition of local infection [3].

Statistical analysis

Statistical analyses were performed using IBM SPSS Statistics (IBM Corp., Armonk, NY, USA). We applied the Student’s t-test for continuous variables and Fisher’s exact test for categorical outcomes to determine the significance of differences observed between the periods before and after the initiation of the pediatric CVAD surveillance and rounding team. The threshold for statistical significance was set at p ≤ 0.05.

## Results

The study included 28 patients before and 39 patients after implementing the rounds. The mean age was 6.9 years (range, 0-15) before and 5.8 years (range, 0-15) after the rounds, with a p-value of 0.36. Similarly, the average observation time was 5.7 months (range, 0.5-16) before and 6.9 months (range, 0.5-19) after the rounds, with a p-value of 0.19, suggesting no significant difference. The diagnoses of patients are shown in Table 2.

**Table 2:** Summary of the diagnoses

	before (n = 28)	after (n = 39)
Leukemia	18 (64.3%)	31 (79.5%)
Malignant lymphoma	3 (10.7%)	4 (10.3%)
Neuroblastoma	1 (3.6%)	1 (2.6%)
Kidney tumor	1 (3.6%)	0 (0%)
Liver tumor	1 (3.6%)	0 (0%)
Brain tumor	2 (7.1%)	0 (0%)
Germ cell tumor	1 (3.6%)	1 (2.6%)
Langerhans cell histiocytosis	1 (3.6%)	2 (5.1%)

There were statistically significant reductions in both the number of dislocations, from eight before (28.6%) to zero (0%) after the rounds (p = 0.001), and in local infections, from five before (17.9%) to one after (2.6%) (p = 0.04). While not statistically significant, the observed reductions in thrombosis, breakage/rupture, and CRBSI suggest a trend toward improvement (see Table 3).

**Table 3:** CVC-related complications CRBSI, catheter-related bloodstream infection

	before (n = 28)	after (n = 39)	p-value
CRBSI	4 (14.3%)	4 (10.3%)	0.45
local infection	5 (17.9%)	1 (2.6%)	0.04
Thrombosis	4 (14.3%)	1 (2.6%)	0.09
Dislocation	8 (28.6%)	0 (0%)	0.001
Breakage/rupture	4 (14.3%)	1 (2.6%)	0.09

## Discussion

The high incidence of CVAD-related complications remains a significant problem in managing pediatric cancer patients, and it underscores the necessity for research focused on preventing and managing these complications among healthcare professionals [1]. There are various types of CVADs, including totally implantable venous access ports, Hickman-Broviac® catheters, non-tunneled catheters, and peripherally inserted central catheters; however, in our hospital, we uniformly use the Hickman-Broviac® catheter for pediatric cancer patients. The Hickman-Broviac® catheter, an external type of CVAD, involves creating a subcutaneous tunnel and placing a cuff within it. This cuff then becomes fibrosed, thereby improving infection defense and fixation [4,5]. The totally implantable venous access port is designed to be completely embedded under the skin. On the other hand, the Hickman-Broviac® catheter has an external exit site, which means it does not require punctures for access. However, this design also increases the risk of catheter dislocation. Therefore, for active infants, methods of insertion from over the shoulder to the back have also been reported to prevent self-removal [2]. This study stands out because of its approach, diverging from those focusing solely on surgical techniques or device selection; instead, it emphasizes the impact of routine rounding and a team-based problem-solving strategy without the introduction of new surgical techniques or fixation method innovations. This approach has led to measurable improvements in patient outcomes, underscoring the importance of team intervention in the overall care process. The establishment of a pediatric CVAD surveillance and rounding team at our hospital is an important step toward improving the quality of care for pediatric cancer patients who require CVADs. The results of this study suggest a significant reduction in CVAD-related complications, specifically the incidence of dislocations and local infections, marking a notable improvement in pediatric CVAD management.

The success of the pediatric CVAD surveillance and rounding team underscores the value of multidisciplinary collaboration in healthcare. The diverse expertise of pediatric surgeons, pediatricians, nurses, nurse practitioners, and ward nursery teachers facilitates comprehensive care that addresses various aspects of CVAD management. This collaborative approach not only enhances problem-solving skills but also promotes a culture of continuous learning and improvement. The effectiveness of multidisciplinary teams in healthcare, particularly in pediatrics, has been demonstrated in several studies [6-8]. These teams improve outcomes for pediatric patients by providing comprehensive care that combines the expertise of different healthcare professionals. Within these teams, improved communication, collaboration, and decision-making processes have positively impacted patient care, including care for immunologic and hemato-oncologic pediatric patients, effectively overcoming teamwork challenges in healthcare settings [6].

The reduction in CVAD-related complications can also be attributed to the emphasis on education and training within the multidisciplinary team. Pediatric residents are given the opportunity to observe CVAD placement procedures, which is likely to improve their understanding and competence in the management of these devices. This focus on education raises questions about the specific training methods used and their effectiveness. Further research could evaluate the impact of simulation-based training, hands-on workshops, and other educational strategies on improving CVAD management skills among healthcare professionals.

While the current study focuses on the professional management of CVADs, the role of patients and their caregivers cannot be overlooked. It is critical to educate patients and their caregivers about CVAD care, potential complications, and how to respond to problems, especially given the difficulty of ensuring young children’s understanding and cooperation in their own care. With this in mind, in addition to appropriate communication during rounds, we have repeatedly educated patients and their caregivers about the importance of CVAD management.

The results of this study provide a solid foundation for further research to improve CVAD care in pediatric populations. Future studies could examine the long-term outcomes of implementing a pediatric CVAD surveillance and rounding team, including the impact on hospital length of stay, ICU admissions, and overall patient satisfaction.

Limitations of the study include its retrospective design and small single-center sample, which may not accurately represent broader patient populations and introduce potential biases.

## Conclusions

Establishing a pediatric CVAD surveillance and rounding team marks a significant advancement in managing CVADs for pediatric cancer patients. The multidisciplinary approach not only reduces complications but also fosters a culture of continuous improvement and patient-centered care. By delving into the diverse perspectives discussed, healthcare professionals can further refine and enhance strategies for managing CVADs in pediatric patients, ultimately leading to better outcomes and quality of life for these patients.
